# The Effect of Speech Variability on Tonal Language Speakers’ Second Language Lexical Tone Learning

**DOI:** 10.3389/fpsyg.2018.01982

**Published:** 2018-10-23

**Authors:** Kaile Zhang, Gang Peng, Yonghong Li, James W. Minett, William S-Y. Wang

**Affiliations:** ^1^Department of Chinese and Bilingual Studies, The Hong Kong Polytechnic University, Hong Kong, China; ^2^Shenzhen Institutes of Advanced Technology, Chinese Academy of Sciences, Shenzhen, China; ^3^Key Lab of China’s National Linguistic Information Technology, Northwest University for Nationalities, Lanzhou, China; ^4^Department of Electronic Engineering, The Chinese University of Hong Kong, Hong Kong, China

**Keywords:** speech variability, the second language acquisition, lexical tones, Cantonese, Mandarin

## Abstract

Speech variability facilitates non-tonal language speakers’ lexical tone learning. However, it remains unknown whether tonal language speakers can also benefit from speech variability while learning second language (L2) lexical tones. Researchers also reported that the effectiveness of speech variability was only shown on learning new items. Considering that the first language (L1) and L2 probably share similar tonal categories, the present study hypothesizes that speech variability only promotes the tonal language speakers’ acquisition of L2 tones that are different from the tones in their L1. To test this hypothesis, the present study trained native Mandarin (a tonal language) speakers to learn Cantonese tones with either high variability (HV) or low variability (LV) speech materials, and then compared their learning performance. The results partially supported this hypothesis: only Mandarin subjects’ productions of Cantonese low level and mid level tones benefited from the speech variability. They probably relied on the mental representations in L1 to learn the Cantonese tones that had similar Mandarin counterparts. This learning strategy limited the impact of speech variability. Furthermore, the results also revealed a discrepancy between L2 perception and production. The perception improvement may not necessarily lead to an improvement in production.

## Introduction

Talkers’ speech exhibits a great deal of acoustic-phonetic variability due to physiological and psychological factors. Even for the same talker, the speech signal may vary substantially in different conditions (Johnson and Mullennix, 1997). Speech perception is slower and less accurate for HV speech due to the lack of trial-by-trial consistency and predictability in the phonetic features (Nusbaum and Morin, 1992; Wong and Diehl, 2003; Peng et al., 2012). However, different from its negative impact on speech perception, speech variability generally facilitates L2 acquisition (e.g., Wang et al., 1999; Perrachione et al., 2011; Sadakata and McQueen, 2014).

The effect of speech variability on improving L2 acquisition is first reported in the learning of segmental components (i.e., consonants and vowels). Lively et al. (1993) trained Japanese speakers to differentiate the English /r/-/1/contrast. Subjects who were trained with multiple talkers’ pronunciations responded faster and more accurately in the post-test compared to subjects trained with tokens from a single talker. Furthermore, multiple-talker training, but not single-talker training, also enabled subjects to generalize the contrasts to novel words and to unfamiliar talkers. In the identification of English /i/ and /I/, Finnish learners of English relied more on duration cues than native speakers who mainly relied on spectral cues. However, after HV training, Finnish learners achieved a more native-like perceptual cue weighting (Ylinen et al., 2010). L2 production is also affected by the variability of training materials. Japanese learners’ production of English /r/ and /1/ was significantly improved after multiple-talker training (Bradlow et al., 1997). English speakers’ production of Russian consonant clusters was more stable and accurate if the training materials were highly variable (Davidson et al., 2015). Apart from the learning of phonological categories, speech variability has also been found to facilitate the acquisition of morphemes, grammar, and words (Barcroft and Sommers, 2005; Torkildsen et al., 2013; Eidsvåg et al., 2015).

Except for the segmental components, lexical tones are also important for L2 learners who want to acquire a tonal language, since lexical tones are used to distinguish lexical meanings in tonal languages (Yip, 2002). For instance, the base syllable /ma/ in Mandarin means “mother” with a high level tone, but means “hemp” with a rising tone. There are two types of lexical tones: contour tones and level tones. Contour tones change their pitch heights over the time course of syllables, whereas the heights of level tones remain relatively steady (Yip, 2002). Several acoustic parameters, such as fundamental frequency (F0), intensity profile, duration, and voice quality, contribute to lexical tone perception (Zhang et al., 2012). Among all these cues, F0, as the primary acoustic correlate of lexical tones, has the greatest importance in lexical tone categorization (Wang, 1972; Bishop and Keating, 2012; Zhang et al., 2012). The two attributes of pitch trajectory (i.e., the F0 trajectory), pitch slope and pitch height, are weighted unequally in perceiving different lexical tones. The differentiation of lexical tones with different pitch contours mainly relies on the pitch slope, whereas, pitch height is critical for the differentiation of tones with similar pitch contours (Peng et al., 2012).

Considering that HV training was effective in improving the acquisition of both segmental components (i.e., consonants and vowels) and larger linguistic units (e.g., morphemes and words), Wang et al. (1999), Perrachione et al. (2011), Heinzen (2014), and Sadakata and McQueen (2014) applied HV training method to L2 lexical tone learning. Wang et al. (1999) reported that multiple-talker speech input significantly improved English speakers’ learning of Mandarin tones and that the improvement was retained 6 months after training. Except for the talker variability, variabilities in the target acoustic dimensions are also helpful (Heinzen, 2014). The naturally produced Mandarin syllables were exaggerated in the syllable duration, pitch height, and pitch range to form the infant-directed speech. After 2–3 h of perceptual training, American English-speaking learners showed significant improvement in the Mandarin tone identification (Heinzen, 2014). However, without a LV condition for comparison, Wang et al. (1999) and Heinzen (2014) could attest only the efficiency of training, not the superiority of HV speech input in comparison to LV speech input. This problem was solved in Perrachione et al. (2011) who trained English speakers to learn an artificial language containing three simple tones, comprising level, rising, and failing tones, with speech produced by either a single or multiple talkers. The results suggested that multiple-talker speech yielded a better learning achievement than the single-talker condition. However, only learners of high perceptual ability (HPA) could benefit from the speech variability, whereas, for learners who were poor at pitch perception, the HV speech input impaired their L2 lexical tone learning compared with the LV speech input. Sadakata and McQueen (2014) extended the work of Perrachione et al. (2011) to the acquisition of a natural language’s tone system. They trained Dutch speakers to learn Mandarin tones with speech of low, medium, or high variability. The results showed that increased variability improved the performance of high-aptitude perceivers but it impeded the low-aptitude perceivers’ Mandarin tone learning.

Learners’ L1 backgrounds are also frequently reported to affect L2 lexical tone learning. The perceptual assimilation model (PAM; Best, 1995) claims that learners tend to assimilate the L2 phonological categories into their L1 phonological categories. The discrimination of two L2 contrasts is poor when they are perceived as belonging to a single L1 category, but excellent when they are perceived as two separate L1 categories. So and Best (2010, 2014), and Cooper and Wang (2012) found that PAM could be used to explain L2 lexical tone acquisition. Cantonese speakers frequently confused the Mandarin high falling tone with the Mandarin high level tone since these two tones were similar to the allophones of the Cantonese high level tone (So and Best, 2014). Thai speakers were usually successful in identifying the Cantonese low falling and low level tone because these two tones could be perceptually mapped to the falling tone and low level tone in Thai, respectively (Cooper and Wang, 2012). Consequently, PAM has been extended to the perceptual assimilation model for suprasegmentals (PAM-S; So and Best, 2010, 2014). Like PAM, the speech learning model (SLM; Flege, 1995, 2007) also emphasizes the influence of the L1 phonological system on L2 learning. SLM holds that all L1 and L2 phonetic elements exist in a common phonological space and mutually influence one another. It predicts that, if an L2 sound differs insufficiently from its phonetically closest L1 sound, a separate mental representation of this L2 phonological category will not be formed. Only when the distance between the L2 sound and its nearest L1 sound is large enough in the common phonetic space will a new category of this L2 sound be constructed. Although the SLM makes no specific prediction about the learning of lexical tones, it is possible that, just as PAM, SLM can be extended to the acquisition of suprasegmentals as well.

Although speech variability has been shown to be effective in improving L2 lexical tone learning (e.g., Perrachione et al., 2011; Sadakata and McQueen, 2014), there are still some open questions. Perrachione et al. (2011), and Sadakata and McQueen (2014) investigated the learning of lexical tones by non-tonal language (English or Dutch) speakers. However, some learners have already mastered a tonal system in their L1s, for example Mandarin learners of Cantonese. It remains unknown how speech variability affects tonal language speakers’ learning of L2 lexical tones. Since L2 acquisition is affected by the L1 background, it is possible that the effect of speech variability on tonal language speakers’ L2 lexical tone learning is different from how it affects the L2 learning of non-tonal language speakers. L2 lexical tones are novel for non-tonal language speakers, and thus they need to form new mental representations for all tone categories. However, the situation for tonal language speakers is complex. Some tone categories in L2 are notably different from the lexical tones in their L1. Based on SLM, leaners probably treat these tones as new tone categories and mental representations for these tones can be established. But some L2 lexical tones may also exist in their L1 or share great similarities with the tones in their L1. As PAM-S and SLM suggest, subjects tend to assimilate these L2 tones into their L1 tone categories and acquire these L2 tones based on the mental representations in their L1. The acquisition of these two types of L2 lexical tones (i.e., different vs. similar) may benefit unequally from the HV training.

There are several reasons why HV training shows superiority in language learning. HV speech highlights which cues are crucial for phonological contrasts and demonstrates a wider range that is allowed for these cues to vary as well (for more details, see Davis, 2015). HV speech enhances learners’ sensitivity to the primary between-category acoustic difference without raising their sensitivity to the within-category acoustic differences (Shinohara and Iverson, 2018). Davis (2015) found that the HV training was useful for learners of low L2 proficiency but not for highly proficient L2 learners because the proficient L2 learners had already acquired this part of knowledge provided by HV training (i.e., the crucial perception cues and the range allowed for variation) from their previous exposure in L2. Therefore, it seems that speech variability is helpful for establishing new categories, but once learners have other strategies available, like previous phonological knowledge, HV training may loss its superiority (Davis, 2015).

Based on the above-mentioned studies (Flege, 1995, 2007; So and Best, 2010, 2014; Davis, 2015), it can be hypothesized that speech variability only facilitates the tonal language speakers’ learning of tones that are different from their L1s, since they have no prior knowledge about these unfamiliar tones. But for tones that are similar to their L1s, the HV training may not outperform the LV training because the learners probably rely on the mental representations of tones in their L1 to learn the similar L2 tones. The interaction between the perceptual cue weightings in L1 and L2 tonal systems is also worth investigation. Francis et al. (2008) found that non-tonal language speakers were more sensitive to the changes of pitch height but tonal language speakers, like Mandarin speakers, put more weight on the pitch direction (i.e., pitch slope). Multiple-talker training effectively shifted non-tonal language speakers’ perceptual cue weighting from pitch height to pitch direction (Chandrasekaran et al., 2010). Following the assumption that speech variability only facilitates the acquisition of new items, it is reasonable to hypothesize that HV training probably affects little on the perceptual cue weighting of tonal language speakers whose L1 and L2 use the same cues to signal pitch contrasts. However, HV training should increase tonal language speakers’ weighting on the cues that are not used in their L1.

By investigating Mandarin speakers’ acquisition of Cantonese tones, the present study aims to shed light on how speech variability affects tonal language speakers’ L2 lexical tone learning. Specifically, the present study wants to test the hypothesis that speech variability only promotes the tonal language speakers’ acquisition of lexical tones and the perceptual cues that are different from those in their L1s. Both Mandarin and Cantonese are tonal languages, but the Cantonese tonal system is more complex. Cantonese has six lexical tones in open syllables: high level Cantonese tone (CT) 55, high rising CT25, middle level CT33, low falling CT21, low rising CT23, and low level CT22, whereas Mandarin only has four lexical tones: high level Mandarin tone (MT) 55, high rising MT35, low falling-rising MT214 (being realized as MT21 at non-final positions in the continuous speech and when the following tone is not a falling-rising tone), and high falling MT51 (Chao, 1930; Yip, 2002). Based on the acoustic similarities revealed by Peng (2006), CT55 should be categorized as MT55, CT25 as MT35, and CT21 as MT21. However, there are no direct counterparts for CT33, CT23, and CT22 in Mandarin. As for the perceptual cues, four Mandarin tones each have different pitch contours and thus their differentiation mainly relies on pitch direction. However, both pitch direction and pitch height are important in Cantonese tone identification, since some of the six tones differ in pitch direction but some, especially three level tones (i.e., CT22–CT33–CT55) differ mainly in pitch height. Based on the hypothesis, HV training was supposed to show advantage in the acquisition of CT33, CT23, and CT22, but not in CT55, CT25, and CT21, and Mandarin learners trained with HV speech would become more sensitive to pitch height than those trained with LV speech. To obtain a comprehensive understanding about how speech variability affects L2 lexical tone learning, the present study included both Cantonese tone production and perception. A speech shadowing paradigm was adopted to train Mandarin speakers’ Cantonese tone production and perception simultaneously (Marslen-Wilson, 1985). Mandarin speakers were trained with either HV or LV speech materials, and then were tested to see if their learning results were allied with our hypothesis.

## Materials and Methods

### Participants

Thirty-five right-handed native Mandarin subjects from Northern China were paid to participate in the experiment. They were randomly divided into two groups based on their training materials: 17 for the HV group and 18 for the LV group. All the participants were either undergraduates or postgraduates, with no self-reported visual, audio, or cognitive deficits. The Mandarin subjects had not received professional training in linguistics, psychology, or music, and were naïve to Cantonese. In addition, 17 native Hong Kong Cantonese speakers were recruited as the control group. The criteria for choosing the Cantonese subjects were the same as those for the Mandarin subjects. The experiment was approved by the Joint Chinese University of Hong Kong – New Territories East Cluster Clinical Research Ethics Committee. Informed written consent was obtained from all participants before the experiment.

### Materials

Twelve Hong Kong Cantonese speakers (six males) were recruited to make recordings in a sound-attenuated booth. These informants did not participate in the following experiments. They were asked to pronounce 36 Cantonese syllables (Table 1) covering the six Cantonese long tones (i.e., lexical tones in open syllables) 10 times in a natural way. Only recordings of good clarity and stability were used in the experiment in order to generate stimuli of high voice quality. Based on this criterion, five speakers’ recordings were selected, with four speakers’ recordings (two males and two females) used for the training stimuli and one speaker’s recordings (one female) used for the test stimuli.

**Table 1:** Thirty-six Cantonese tonal syllables.

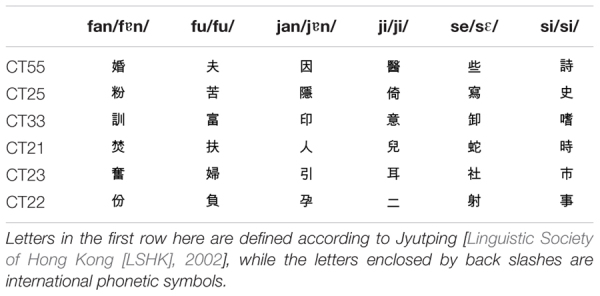

There were two types of training materials: HV training stimuli and LV training stimuli. The HV training stimuli comprised speech with HV in both pitch height and pitch slope. The LV training stimuli comprised speech with LV in both pitch height and pitch slope. For the HV training stimuli, four samples were selected from the 10 repetitions of each tonal syllable produced by each selected speaker (4 speakers × 36 tonal syllables × 4 samples selected from the 10 repetitions). To minimize the variability of the LV training stimuli, only one sample was chosen and reduplicated four times (4 speakers × 36 tonal syllables × 1 sample from the 10 repetitions × 4 reduplications). These samples were also chosen based on clarity and stability.

The pitch heights of the selected stimuli were further manipulated. Each stimulus of the HV training materials was adjusted to a pitch height chosen randomly from the numbers listed in the second row of Table 2 (the HV pitch heights) according to its tonal category. The pitch height manipulation was also carried out for the LV training materials, and the adjustment was based on the LV pitch heights (the third raw in Table 2). The HV and LV pitch heights have the same medians, but different ranges, with a 0.5 or 0.3 pitch height range for HV stimuli and a 0.1 pitch height range for LV stimuli. The HV pitch height range of 0.3 for CT33 and CT22 was chosen so that the categorical boundaries of these two tones did not overlap. The medians of the pitch heights were obtained by referring to the grand mean pitch heights, which were calculated according to the following procedures. First, the raw F0 values of each utterance were analyzed with Praat (Boersma and Weenink, 2014) and transformed from Hertz to log-scale 5-level values (Peng and Wang, 2005). Based on the log-scale 5-level values, the mean pitch height of each utterance was calculated. Only the middle 80% of the time-course of each F0 trajectory was used in order to decrease the tone-irrelevant variation (Peng, 2006). Then, the grand mean of the pitch height of each tonal category was obtained by averaging all 12 informants’ productions of the same tone category. Twelve, instead of five, informants’ recordings were used to obtain the grand means so that the values obtained were closer to the population means. Based on the grand mean pitch heights, the present study set the median for the pitch heights of each tonal category as follows: 4.75 for CT55, 3 for CT25, 3.25 for CT33, 1.75 for CT21, 2.5 for CT23, and 2.75 for CT22.

**Table 2:** The pitch heights used to manipulate the stimuli and the grand mean pitch height of each tonal category.

Tone category	CT55	CT25	CT33	CT21	CT23	CT22
HV pitch heights	4.75 ± 0.25	3 ± 0.25	3.25 ± 0.15	1.75 ± 0.25	2.5 ± 0.25	2.75 ± 0.15
LV pitch heights	4.75 ± 0.05	3 ± 0.05	3.25 ± 0.05	1.75 ± 0.05	2.5 ± 0.05	2.75 ± 0.05
Grand mean pitch height	4.583	2.746	3.256	1.945	2.414	2.757

**FIGURE 1:**
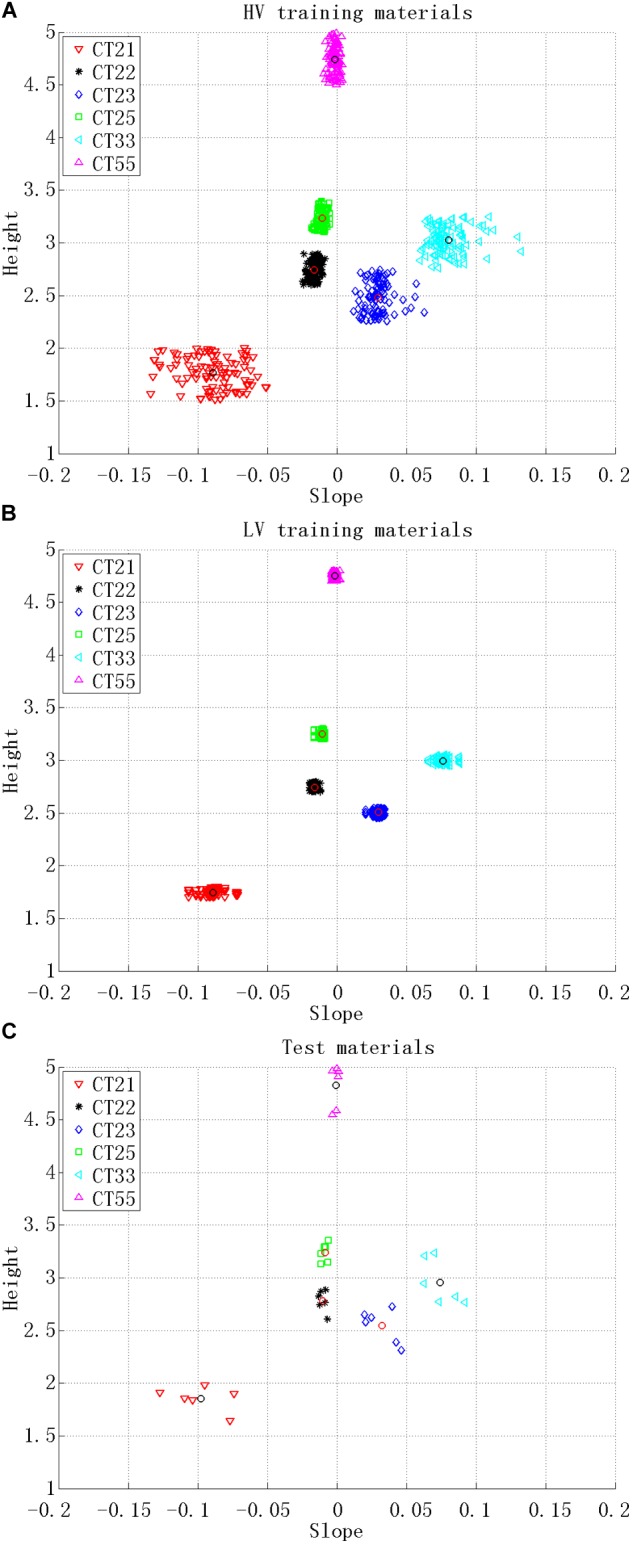
The tone charts for the auditory stimuli used as: **(A)** HV training materials, **(B)** LV training materials, and **(C)** test materials. Height represents the pitch height and Slope represents the pitch slope. Each point in the figure represents a single stimulus. Six tone categories are distinguished by different shapes (also in different colors online) as shown in the legend. The grand mean of the 12 informants’ productions of each tone category is represented by either a black or red circle within a tone category.

Regarding the test stimuli, only one sample (with the best voice quality) was chosen from the 10 repetitions of each tonal syllable (1 speaker × 36 tonal syllables × 1 sample out of 10 repetitions). Their pitch heights were adjusted based on the HV pitch heights. Finally, 1152 stimuli were used as training materials, comprising 576 stimuli as the HV training materials (Figure 1A), 576 stimuli as the LV training materials (Figure 1B), and 36 stimuli as the test materials (Figure 1C).

### Procedure

**FIGURE 2:**
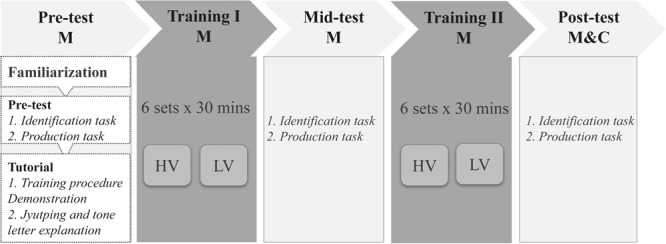
The flow chart of the experimental design. The abbreviation M stands for Mandarin subjects, C for Cantonese subjects, HV for the high variability training group and LV for the low variability training group. The tests were conducted at lab and the trainings were finished at home.

Figure 2 illustrated the experimental design. Mandarin subjects participated in two sessions of the Cantonese tone training and three sessions of the Cantonese tone tests. The tests were carried out before the first training session (pre-test), between the two training sessions (mid-test), and after the second training session (post-test). Cantonese controls only took part in one test session.

#### The Training Sessions

The whole experiment consisted of two training sessions, one after the pre-test and another after the mid-test. Each training session comprised six training sets and Mandarin subjects were asked to finish one training set every 2 days. Each training set consisted of 576 stimuli (4 speakers × 36 tonal syllables × 4 samples). Stimuli from the same speaker (144 stimuli) were blocked, resulting in four blocks in each training set. The 36 tonal syllables were played sequentially (i.e., /fɐn55/, /fɐn33/, /fɐn22/, /fɐn21/, /fɐn23/, /fɐn25/, /fu55/, …) within each block. Mandarin subjects were randomly assigned to either the HV or LV training materials and were asked to finish the training at a quiet place of their own choice using their own computers and headphones. It took about 30 min to finish one training set. Subjects received 3 h training in each session and the total amount of training in the present study was around 6 h which was longer than most of the previou studies (e.g., Wang et al., 1999; Heinzen, 2014; Sadakata and McQueen, 2014).

The speech shadowing paradigm was adopted to train Mandarin subjects’ Cantonese tone perception and production simultaneously (Marslen-Wilson, 1985). The stimulus presentation for training was controlled by Praat (Boersma and Weenink, 2014). The experimenters demonstrated to each participant how to do training with Praat after the pre-test. Subjects were first asked to adjust to a volume level which allowed them to perceive the audio stimuli clearly. In each trial, a training stimulus was played to the subjects. The corresponding traditional Chinese character, its Jyutping transcription [Linguistic Society of Hong Kong [LSHK], 2002], and the tone letter (Chao, 1930) of each stimulus were also shown on the screen for the learners’ reference. When the training started, they were instructed to pay attention to the sound stimulus they heard, especially the pitch, and the visual information on the screen, especially the tone letter which indicated its tone category. They were required to immitate the word as accurately as possible after a stimulus was played. A brief tutorial about how to read Cantonese Jyutping transcription and tone letters was also delivered to each participant by the experimenters after the pre-test. Each participant received a timetable which reminded him/her of the dates for trainings and tests. During training, the Praat script recorded subjects’ productions simultaneously in Waveform Audio File Format. Subjects were asked to send their recordings once they finished their training on that day. The recordings were checked by the experimenters to make sure that the participants followed the instructions well to do the training. Participants who failed to do so were excluded from the present study immediately.

#### The Test Sessions

Each test session compromised two tasks: the production task and the identification task. Before each test, subjects received written instructions and became familiarized with the experiment via a practice session. The auditory stimuli used in the practice session were different from those used in the tests. Stimulus presentations were controlled by E-Prime 2.0 in the test sessions. Since all Mandarin subjects had no prior knowledge of Cantonese, a 15-min perception and production training was provided immediately before the two tasks in the pre-test. The procedure of the 15-min training was almost the same as the one described in “The Training Sessions.” But this short training contained only 288 trials (2 speakers × 36 tonal syllables × 4 samples).

##### The Cantonese tone production task

Subjects’ productions were recorded with Adobe Audition in a sound-insulated booth. The characters used in the production task were the same as those used in training (Table 1). In each trial, a traditional Chinese character, together with the corresponding Jyutping transcription and tone letter, was presented on the screen. Subjects were instructed to read aloud the characters as naturally as possible, and were encouraged to correct or repeat their pronunciations whenever necessary. Each production task consisted of 108 trials (36 characters × 3 repetitions), which were mixed and played in a random order. After finishing 54 trials, subjects could take a 1-min break. This was a self-paced production task. Once subjects were satisfied with their pronunciation for each trial, they were required to press the space button to move on to the next trial.

##### The Cantonese tone identification task

**FIGURE 3:**
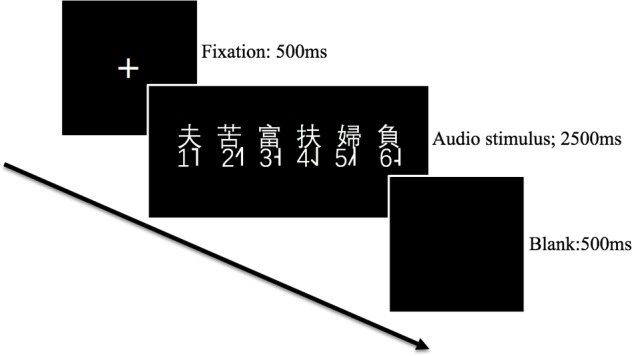
The trial procedure of the identification task.

The test stimuli (36 tonal syllables × 5 repetitions) were mixed and played in a random order. The trial procedure is illustrated in Figure 3. In each trial, an auditory stimulus was played binaurally to subjects, and six traditional Chinese characters sharing the same base syllable but having distinct lexical tones were also shown on the screen at the same time. Subjects were instructed to press a button from 1 to 6 on the keyboard (see Figure 3) to indicate which tone they perceived. The maximum allowable response time was 2500 milliseconds (ms).

### Data Analysis

The present study first compared subjects’ overall performance in the three tests (i.e., pre-, mid-, and post- test) to see whether subjects’ performance after training (i.e., in the mid- and post- tests) was significantly different from their performance in the pre-test. We then analyzed subjects’ learning improvement after each training session to see whether subjects trained with different speech materials (HV vs. LV) showed significantly different learning improvement. Because the effect of speech variability on L2 lexical tone learning has been reported to be constrained by subjects’ perceptual abilities (Perrachione et al., 2011; Sadakata and McQueen, 2013, Sadakata and McQueen, 2014), the present study also took this factor into consideration while analyzing both production and perception data. The perceptual ability in Perrachione et al. (2011) refers to the pretraining aptitudes to perceive pitch contours. Perrachione et al. (2011) asked subjects to identify whether the pitch contour embedded in the vowel was level, falling or rising. Subjects whose accuracy rates were higher than 70% were classified as leaners with strong perceptual abilities. Sadakata and McQueen (2013) measured this ability via the discrimination of lexical tones in the pre-test. Sadakata and McQueen (2014) evaluated subjects’ perceptual aptitudes based on their categorical perception of lexical tones. High-aptitude perceivers’ identification curves were steeper (i.e., the identification slope coefficients were less than 0.2). As can be seen, the perceptual ability in these studies essentially refers to subjects’ ability to differentiate or classify lexical tones. Therefore, the present study divided Mandarin subjects into two groups: learners with HPA and learners with low perceptual ability (LPA) based on the results of the Cantonese lexical tone identification task in the pre-test.

#### Production Data

Subjects’ productions were analyzed acoustically. The pitch trajectories of all the recordings obtained during the production task were characterized by two variables: the pitch height and the pitch slope. The procedure to obtain the pitch height and the pitch slope was the same as the procedure described in “Materials”. The native norm for each tonal category was calculated by averaging 29 native Cantonese speakers’ productions (12 informants recruited for speech recording and 17 subjects recruited as the control group) across all pronunciations with the same tone. The acoustic distance between each utterance (corresponding to a syllable) produced by Mandarin subjects and the native norm was calculated according to the following formula:

(1) $$\begin{array}{c} D=\sqrt{{\left(H_{m}-H_{c}\right)}^{2}+{\left(S_{m}\times 10-S_{c}\times 10\right)}^{2}} \end{array}$$

where *H_m_, S_m_, H_c_*, and *S_c_* represent the pitch height and the pitch slope of Mandarin subjects’ utterances and the pitch height and the pitch slope of the native norm, respectively. The pitch slope, ranging between -0.2 and 0.2, was multiplied by 10 so that it was enlarged to the same range as the pitch height (1–5; see Figure 1). Smaller distance means higher resemblance between a Mandarin subjects’ production and the native norm. The production improvement after the first training session was calculated as the mid-test distance minus the pre-test distance, and the improvement after the second training session was calculated as the post-test distance minus the mid-test distance (Bradlow et al., 1997). If subjects’ production improved after training, the production improvement would be a negative number. Therefore, the smaller the number obtained, the bigger the production improvement.

#### Perception Data

In each trial, a tonal syllable was played to subjects. If subjects could correctly identify the tonal category of the stimulus they heard, the trial was counted as a correct one. Otherwise, it was regarded as wrong. Subjects’ perception accuracies in all three test sessions were calculated. The perception improvement after the first training session was defined as the mid-test accuracy minus the pre-test accuracy. Likewise, the perception improvement after the second training session was the post-test accuracy minus the mid-test accuracy (Bradlow et al., 1997). To further illustrate the detailed perceptual results, the confusion matrices of the identification task are also included. The confusion matrix shows how frequently a target tonal category is identified as each of the six Cantonese tone categories (Wang et al., 1999).

## Results

Subjects were divided into two groups – HPA group and LPA group – based on the results of the identification task in the pre-test. The independent *t*-test suggested that the perception accuracy of HPA group (18 subjects; *M* = 0.7, *SE* = 0.04) was significantly higher than the accuracy of the LPA group (*M* = 0.55, *SE* = 0.07), *t*(33) = -8.137, *p* < 0.01.

### The Cantonese Tone Production Task

**FIGURE 4:**
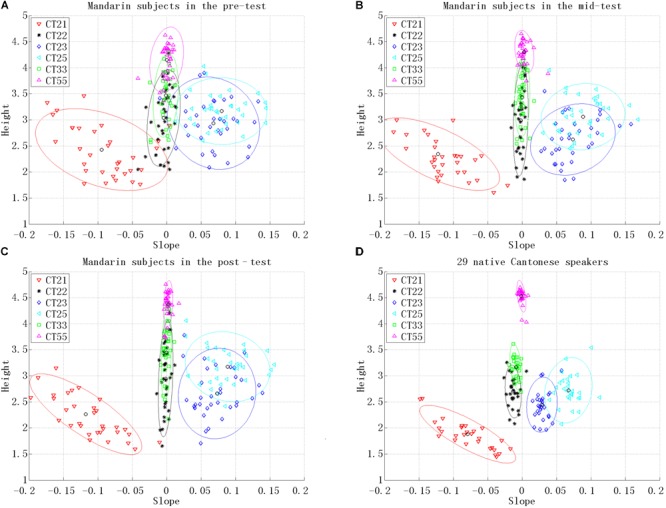
The tone charts for: **(A)** Mandarin subjects in the pre-test, **(B)** Mandarin subjects in the mid-test, **(C)** Mandarin subjects in the post-test, and **(D)** 29 native Cantonese speakers. The six tone categories are represented by different shapes (also in different colors online) as shown in the legend.

An overall view of Mandarin subjects’ and Cantonese subjects’ Cantonese tone production results is shown in Figure 4. Each point in the charts represents the average value of a single subject’s production of one tone category. The ellipses were drawn to cover 90% of the points belonging to the same tone category. The tone production data of Mandarin subjects showed greater variation than those of Cantonese subjects. The three level tones produced by Mandarin subjects overlapped substantially, as did the two rising tones. This was also the case for Cantonese subjects, but to a lesser degree, with clearer differentiation between tonal categories than for Mandarin subjects. Even though Mandarin subjects’ productions were notably different from those of native Cantonese speakers, their improvement could be observed across the three test sessions. First, the reduction in the areas of the ellipses indicated that Mandarin subjects’ productions of each tonal category became less varied. Moreover, the distinction between the two rising tones became clearer after training, and CT55 was seldom confused with the two other level tones at post-test. The distances between the corresponding native norms (Table 3) and utterances of Mandarin subjects were averaged for each subject over three repetitions and the six base syllables (i.e., 18 tone tokens per tone category per subject). A one-way ANOVA with *test* (pre-test, mid-test, and post-test) as the within-subject factor was conducted on the distances which were further averaged across six lexical tones. The analysis showed that there was significant difference across tests, *F*(2,68) = 12.005, *p* < 0.01. Subjects’ production was significantly closer to the native norm in the mid-test (*M* = 0.664, *SE* = 0.031) compared with the pre-test (*M* = 0.742, *SE* = 0.03), suggesting the effectiveness of training. However, the distance was not reduced significantly in the post-test (*M* = 0.642, *SE* = 0.029) compared with the mid-test.

**Table 3:** The native norm for each Cantonese tone category.

	CT55	CT25	CT33	CT21	CT23	CT22
Pitch height	4.535	2.726	3.170	1.881	2.444	2.700
Pitch slope	–0.001	0.068	–0.009	–0.079	0.030	–0.013

The production improvements after each training session were calculated based on the method mentioned in “Data Analysis.” A four-way repeated measures ANOVA was carried out on the production improvement, with the Greenhouse–Geisser method used to correct for violations of sphericity. The within-subject factors were *training session* (the first training session and the second training session) and *tone* (CT55, CT25, CT33, CT21, CT23, and CT22), and the between-subject factors were *variability* (HV and LV) and *perceptual ability* (HPA and LPA). The analysis revealed a significant *tone* × *variability* interaction, *F*(5,155) = 2.738; *p* < 0.05. The simple main effect analysis on the *tone* × *variability* interaction suggests that HV training was significantly more effective than LV training in improving Mandarin subjects’ learning of CT33 and CT22 (*ps* < 0.05; see Table 4). The HV training and LV training showed no significant difference on the production improvement of other tonal categories (Table 4). The *training session* and the *perceptual ability* factors were not involved in any main effects or significant interactions. No significant *perceptual ability* by *variability* interaction was found, which indicated that the effect of speech variability on improving lexical tone production was not affected by subjects’ perceptual abilities.

**Table 4:** The production improvement of each tone category.

	HV training		LV training		
	Mean	Standard error	Mean	Standard error	*p*
CT55	–0.089	0.029	–0.089	0.027	0.989
CT25	0.002	0.035	–0.04	0.034	0.394
CT33	–0.076	0.029	0.016	0.027	0.026
CT21	–0.059	0.045	–0.065	0.043	0.934
CT23	–0.072	0.04	–0.064	0.038	0.883
CT22	–0.11	0.027	0.049	0.026	<0.01

*The improvements were averaged across the first and second training sessions.*

### The Cantonese Tone Identification Task

**FIGURE 5:**
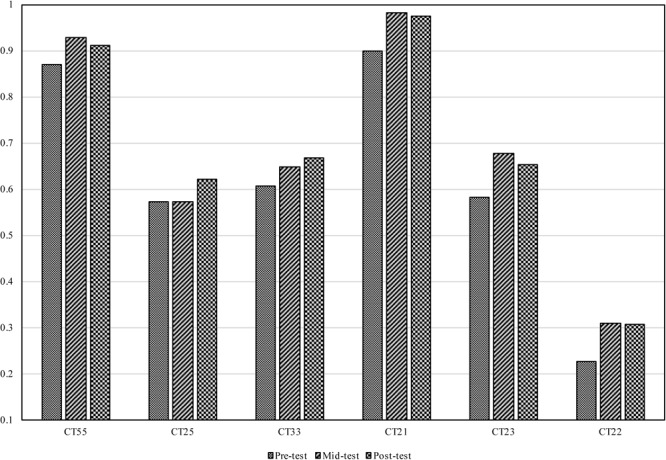
The accuracies for the Cantonese tone identification task in the three tests.

The accuracies of Mandarin subjects’ Cantonese tone identification in the three tests are demonstrated in Figure 5. As Figure 5 suggested, Mandarin subjects showed improvement in all lexical tones. To evaluate whether the accuracies after training (i.e., in the mid- and post- test) were significantly higher than the accuracy in the pre-test, a one-way ANOVA with *test* (pre-test, mid-test, and post-test) as the within-subject factor was conducted on the accuracies which were averaged across syllables and lexical tones. The analysis revealed a significant main effect of *test, F*(2,66) = 29.903, *p* < 0.001. Subjects achieved significantly better results in the mid-test (*M* = 0.687, *SE* = 0.013) compared with the pre-test (*M* = 0.626, *SE* = 0.016). However, no further significant improvement was observed in the post-test (*M* = 0.689, *SE* = 0.015) compared with the mid-test.

The perception improvements were calculated based on the methods mentioned in “Data Analysis”. They were submitted to a four-way repeated measures ANOVA with *training session* (the first training session and the second training session) and *tone* (CT55, CT25, CT33, CT21, CT23, and CT22) as the within-subject factors and *variability* (HV and LV) and *perceptual ability* (HPA and LPA) as the between-subject factors. The Greenhouse–Geisser method was used to correct violations of sphericity. The analysis revealed a main effect of *training session, F*(1,31) = 18.25; *p* < 0.001, and a significant *training session* × *perceptual ability* interaction, *F*(1,31) = 11.28; *p* < 0.05. The simple main effect analysis of the s*ession* × *perceptual ability* interaction with Bonferroni adjustment showed that HPA leaners’ improvement in the Cantonese tone perception after the first training session (*M* = 0.032, *SE* = 0.013) was not significantly different from their perception improvement after the second training session (*M* = 0.019, *SE* = 0.011; *p* = 0.513). However, for LPA learners, the perception improvement after the first training session (*M* = 0.091, *SE* = 0.014) was significantly higher than their improvement after the second training session (*M* = -0.018, *SE* = 0.02, *p* < 0.05). The *tone* and *variability* factors were not involved in any main effects or significant interactions. The analysis of the perception improvement also failed to find a *variability* by *perceptual ability* interaction. It seems that, for tonal language speakers, the impact of speech variability is not constrained by speakers’ perceptual abilities.

The confusion matrices (Table 5) illustrate the detailed Cantonese tone identification results. Both Mandarin and Cantonese subjects were proficient at identifying CT21, the only falling tone, and CT55, the tone with the highest pitch height, but were comparatively poor at differentiating tone pairs with similar pitch contours, particularly CT25-CT23 and CT33-CT22. Cantonese speakers’ accuracy for every tonal category was higher than that of Mandarin speakers, as would be expected. The difference between Mandarin and Cantonese subjects was evident for the level tones, which suggested that Mandarin subjects were not as proficient as Cantonese speakers in estimating the pitch height of isolated level tones. Besides, Mandarin subjects’ perception of Cantonese level tones also showed a significant L1 influence. Mandarin subjects sometimes misperceived CT22 as CT55 (12%), whereas Cantonese subjects seldom did so (1%). Furthermore, Cantonese speakers were more likely to perceive CT33 as CT22 (11%) which was acoustically more similar to CT33. In contrast, Mandarin speakers frequently perceived CT33 as CT55 (24%), a tone that also exists in Mandarin (i.e., MT55).

**Table 5:** The confusion matrices of the identification task for: (A) Mandarin and (B) Cantonese subjects. The confusion matrix of Mandarin subjects was based on the perception results averaged across the mid- and post-tests.

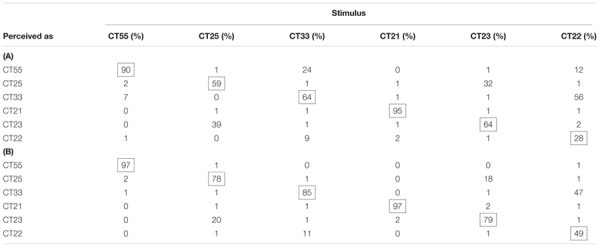

### The Correlation Between the Perception and Production Tasks

The correlation analyses between the perception and production tasks were first conducted on the overall performance. The identification accuracies and the production distances were averaged across six lexical tones to represent the overall performance in each test. The results indicated that the perceptual accuracy was highly and negatively correlated with the production distance in the pre-test, *r* = -0.528, *p* < 0.05, the mid-test, *r* = -0.569, *p* < 0.01, and the post-test, *r* = -0.523, *p* < 0.05. Such results suggest if a Mandarin subject perceive Cantonese lexical tones more accurately, he/she in general produce the tones in a more native-like way.

The production and perception improvements, however, were not significantly correlated after the first training session, *r* = 0.294, *p* = 0.087 or after the second training session, *r* = 0.108, *p* = 0.537. The production and perception improvements of each lexical tone also showed no significant correlation (*p*s > 0.05), suggesting that improvement in one modality may not lead to a similar degree of improvement in another modality.

## Discussion

The comparison between the production distances in the three tests suggested that subjects’ production was improved after training. The manipulation of speech variability only affected Mandarin speakers’ production of CT33 and CT22, but not others, which was partially consistent with the hypothesis of the present study. Training also improved subjects’ Cantonese tone perception. However, the perceptual results were not in line with our prediction. HV training materials showed statistically similar effects on subjects’ perception improvement as the LV training materials, even for CT33, CT23, and CT22 which were distinct from Mandarin tones. Subjects who obtained high accuracies in Cantonese tone perception also showed a more native-like Cantonese tone production. But the degrees of improvements in two modelities (i.e., production and perception) were not correlated. Different from pervious studies (Perrachione et al., 2011; Sadakata and McQueen, 2013, 2014), speech variability did not show an unequal effect on learners with different perceptual aptitudes.

### The Effect of Speech Variability on Mandarin Subjects’ Production of Cantonese Tones

As the hypothesis of the present study predicted, the relationship between the L1 and L2 tonal systems constrains the effect of speech variability on tonal language speakers’ L2 lexical tone acquisition. The comparison of the Mandarin and Cantonese tone inventories in Peng (2006) reveals that three tone pairs: CT55-MT55, CT21-MT21, and CT25-MT35 share great similarities. However, CT22 and CT33 cannot be mapped directly to any Mandarin tone categories. The production results in the present study suggest that speech variability only facilitates the acqusistion of Cantonese tones that are notably different from the native Mandarin tones (i.e., CT22 and CT33). But the variability manipulation of speech input does not affect the production results of Cantonese tones which have acoustically similar counterparts in Mandarin (i.e., CT55, CT21, and CT25).

Mandarin subjects probably employed different learning strategies to acquire different Cantonese tones. As suggested by PAM-S and SLM, learners may rely on the phonological categories in their L1 to acquire the similar phonological categories in L2 (Flege, 2007; So and Best, 2010). Once detecting that CT55, CT21, and CT25 were similar to MT55, MT21, and MT35, respectively, Mandarin subjects may have used the mental representations of MT55, MT21, and MT35 to identify the similar tonal categories in Cantonese. Consequently, regarding the training materials of CT55, CT21, and CT25, they may have paid less attention to their acoustic details, thereby invalidating the impact of the speech variability manipulation. Even if they did attend closely to the training materials, the mental representations of MT55, MT21, and MT35 probably did not change significantly during the short period of training, since these Mandarin tone representations have been formed as a result of long-term exposure. Consequently, the HV and LV trainings could not lead to different learning outcomes given that both HV and LV groups relied on similar, pre-existing native mental representations to learn the corresponding Cantonese tones. However, unlike the acquisition of CT55, CT21, and CT25, Mandarin subjects could not find counterparts of CT33 and CT22 in Mandarin. SLM predicts that, when the distance between a L2 sound and its nearest L1 sound is sufficiently great, formation of a mental representation for a new L2 category will occur (Flege, 2007). Therefore, Mandarin subjects’ construction of mental representations for CT33 and CT22 was heavily reliant on the training materials, allowing the beneficial effect of HV speech to facilitate lexical tone learning. As Davis (2015) suggested, HV speech can draw learners’ attention to the acoustic cues that are meaningful for phonological contrasts and demonstrate a wider range that is allowable for their variation. Besides, it is also believed that HV training enhances subjects’ ability to normalize speech variability, which is important for constructing stable mental representations of level tones. Since the differentiation of Cantonese level tones mainly relies on pitch height, the superiority of HV training on the production of level tones also supports the hypothesis that HV training increases Mandarin subjects’ sensitivity to pitch height, the cue that is comparatively less used in Mandarin tonal system.

Because there is no low rising tone in Mandarin, we hypothesized that the acquisition of CT23 would benefit from speech variability as well. However, the results were not in consistent with our hypothesis. One possible explanation was that Mandarin subjects’ acquisition of CT23 also relied on the mental representation of MT35. Mandarin learners in the present study can hardly differentiate CT25 and CT23 in either their perception (see Table 5A) or production (see Figures 4A–C), suggesting that the acquisition of these two tones might rely on the same mental representation. Peng (2006) plotted the tone charts for Mandarin and Cantonese each based on more than 60 subjects’ production in the continuous speech. The Mandarin tone chart in Peng (2006) suggested that the realization of MT35 varied a lot. Sometimes, it was pronounced as a full MT35 with a steep tone slope and comparatively high pitch height, and sometimes it was pronounced in a reduced form which was acoustically closer to CT23. The comparison between Cantonese and Mandarin tone charts in Peng (2006) further indicated that the distribution of MT35 was largely overlapped with both CT25 and CT23. Therefore, it was possible that Mandarin subjects perceived CT23 as a reduced form of MT35 and relied on the mental representation of MT35 to learn CT23 as well. As a result, the acquisition of CT23 was not significantly affected by the different amount of variation of the training materials, resulting in the comparable learning outcomes under HV and LV training.

### The Effect of Speech Variability on Mandarin Subjects’ Perception of Cantonese Tones

The present study hypothesized that Mandarin subjects trained with HV speech should achieve higher accuracies in identifying CT33, CT22, and CT23. However, HV training showed no superiority in facilitating Mandarin subjects’ perception of any Cantonese tones. The reason why speech variability failed to improve Mandarin learners’ perception of CT55, CT25, CT21, and CT23 is largely the same as for the production. That is, due to the acoustic similarity, Mandarin subjects may rely on the existing mental representations of MT55, MT35, and MT21 while learning CT55, CT25, CT23, and CT21, rendering the variation of training materials invalid. The effects of speech variability on Mandarin subjects’ acquisition of CT33 and CT22 were complex in the present study. The production of CT33 and CT22 benefited from speech variability but the perception of them did not. The improvements in the production of CT33 and CT22 were also not significantly correlated with their improvements in the perception. The asymmetrical results do not support one of the predictions of SLM that speech production is guided by the perceptual representations (Flege, 2007).

It was multifaceted for the reasons why HV training did not outperformed LV training in Mandarin subjects’ perception of CT22 and CT33. One possible reason could be the difficulty in perceiving Cantonese level tones in isolation. The differentiation of Cantonese level tones CT55, CT22, and CT33 relies mainly on pitch height. However, the inter- and intra- talker variability makes the absolute pitch height a less reliable cue. Wong and Diehl (2003), Francis et al. (2006), and Peng et al. (2012) have shown that context is indispensable in the correct identification of Cantonese level tones. The present study asked subjects to identify the tones of isolated speech stimuli. The confusion matrix (Table 5A) showed that Mandarin subjects perceived 56% trials of CT33 as CT22, indicating that they encountered great difficulty in differentiating CT33 from CT22 in isolation. Besides, Mandarin subjects perceived 24% trials of CT33 and 12% trials of CT22 as CT55, which resembled MT55, but Cantonese subjects seldom confused CT33 (0%) or CT22 (1%) with CT55. Such perceptual differences suggested that Mandarin subjects’ perceptions of CT33 and CT22 were also influenced by their L1 experience. The interference from both L1 and L2 made the perception of CT33 and CT22 in isolation a difficult task. The advantage of HV training was not strong enough to enable Mandarin subjects to differentiate CT33 and CT22 in isolation more accurately.

The inefficacy of speech variability could also be partially attributed to the training protocols. Several aspects of the training procedure in the present study were different from those of previous studies which reported the superiority of HV training. One important difference lies in the degree of variation of training materials. Previous studies (Perrachione et al., 2011; Sadakata and McQueen, 2014) which included both HV and LV trainings generally manipulated the number of talkers. The HV training materials consisted of multiple-talker productions, whereas the LV training materials were composed by single-talker productions. Apparently, regarding the degree of variation, the difference between HV training and LV training in Perrachione et al. (2011) and Sadakata and McQueen (2014) was much larger than that in the present study. Heinzen (2014) also included four speakers’ recordings and manipulated the target acoustic dimensions to increase the variation, which were similar to the present study. However, to create the infant-directed speech, Heinzen (2014) exaggerated each acoustic cue by a comparatively larger degree and introduced four levels of exaggeration as well. Therefore, the speech variation in Heinzen (2014) was also larger than the HV speech in the present study. It is possible that the inefficacy of the speech variability manipulation was caused by the comparatively small difference between the HV and LV training materials. Besides, most of the previous studies used the perception training but the training in the present study was a combination of perception and production. Subjects received no feedback about their production trial by trial. It was likely that their non-standard pronunciation without correction hindered their learning.

The present study also revealed a discrepancy between the L2 perception and production. Even though subjects’ performances in L2 perception and production were highly correlated, the speech production may not be guided by the perceptual learning results. The analysis revealed that the improvement in production was not correlated with the perception improvement. The in-depth inspection even found that four Mandarin subjects improved their production but not their perception after two training sessions (Zhang and Peng, 2017). The results that subjects’ production but not their perception of CT33 and CT22 were affected by the speech variability further suggested that the relationship between two modalities was much more complicated than SLM predicts (Flege, 2007).

## Conclusion

Speech variability in the present study showed a comparatively small effect on Mandarin speakers’ learning of Cantonese tones. HV training only facilitated Mandarin subjects’ production of CT33 and CT22, the tones that were not similar to any Mandarin tone categories, but did not promote their production of CT55, CT25, CT23, or CT21, which had similar counterparts in Mandarin. The production results supported the hypothesis that speech variability only facilitated the acquisition of tonal categories which were different from the tones in their L1. The production results also suggested that Mandarin subjects were more sensitive to the pitch height after HV training. However, the superiority of HV training was not shown on the perception of CT22 and CT33. The inefficacy of speech variability might be caused by the difficulty in identifying the pitch height of isolated Cantonese level tones. Besides, the comparatively small variation of the HV training materials might also reduce the effectiveness of HV training. Further studies with a better control on the training protocols are needed to test whether the hypothesis of the present study can also be applied to tonal language speakers’ perception of L2 lexical tones.

## Author Contributions

KZ, GP, YL, JM, and WW designed the experiments. KZ performed the experiments and drafted the manuscript. KZ, GP, and YL analyzed the data. KZ, GP, JM, and WW revised the manuscript.

## Conflict of Interest Statement

The authors declare that the research was conducted in the absence of any commercial or financial relationships that could be construed as a potential conflict of interest.

## References

[B1] Barcroft J. Sommers M. S. (2005). Effects of acoustic variability on second language vocabulary learning. *Stud. Second Lang. Acquis.* 27 387–414. 10.1017/S0272263105050175

[B2] Best C. T. (1995). “A direct realist perspective on cross-language speech perception,” in *Speech Perception and Linguistic Experience: Theoretical and Methodological Issues in Cross-Language Speech Research* ed. Strange W. (Timonium, MD: York) 171–206.

[B3] Boersma P. Weenink D. (2014). *Praat: Doing Phonetics by Computer.* Available at: http://www.praat.org [accessed September 14 2004].

[B4] Bishop J. Keating P. (2012). Perception of pitch location within a speaker’s range: fundamental frequency, voice quality and speaker sex. *J. Acoust. Soc. Am.* 132 1100–1112. 10.1121/1.4714351 22894229

[B5] Bradlow A. Pisoni D. B. Akahane-Yamada R. Tohkura Y. (1997). Training japanese listeners to identify english /r/ and /l/: IV. Some effects of perceptual learning on speech perception. *J. Acoust. Soc. Am.* 101 2299–2310. 10.1121/1.418276 9104031 PMC3507383

[B6] Chandrasekaran B. Sampath P. D. Wong P. C. M. (2010). Individual variability in cue-weighting and lexical tone learning. *J. Acoust. Soc. Am.* 128 456–465. 10.1121/1.3445785 20649239 PMC2921440

[B7] Chao Y. R. (1930). A system of tone-letters. *Le Maître Phonétique* 45 24–27.

[B8] Cooper A. Wang Y. (2012). The influence of linguistic and musical experience on cantonese word learning. *J. Acoust. Soc. Am.* 131 4756–4769. 10.1121/1.4714355 22712948

[B9] Davidson L. Martin S. Wilson C. (2015). Stabilizing the production of nonnative consonant clusters with acoustic variability. *J. Acoust. Soc. Am.* 137 856–872. 10.1121/1.4906264 25698019

[B10] Davis A. K. (2015). *The Interaction of Language Proficiency and Talker Variability in Learning.* Ph.D. thesis, University of Arizona Arizona.

[B11] Eidsvåg S. Austad M. Plante E. Asbjørnsen A. (2015). Input variability facilitates unguided subcategory learning in adults. *J. Speech Lang. Hear. Res.* 58 826–839. 10.1044/2015_JSLHR-L-14-0172 25680081 PMC4610293

[B12] Francis A. L. Ciocca V. Wong N. K. Y. Leung W. H. Y. Chu P. C. Y. (2006). Extrinsic context affects perceptual normalization of lexical tone. *J. Acoust. Soc. Am.* 119 1712–1726. 10.1121/1.2149768 16583914

[B13] Francis A. L. Ciocca V. Ma L. Fenn K. (2008). Perceptual learning of cantonese lexical tones by tone and non-tone language speakers. *J. Phon.* 36 268–294. 10.1016/j.wocn.2007.06.005 25465395

[B14] Flege J. E. (1995). “Second language speech learning: theory, findings, and problems,” in *Speech Perception and Linguistic Experience: Issues in Cross-Language Research* ed. Strange W. (Baltimore, MD: York) 233–276.

[B15] Flege J. E. (2007). “Language contact in bilingualism: phonetic system interactions,” in *Laboratory Phonology 9* eds Cole J. Hualde J. I. (Berlin: Walter de Gruyter) 353–382.

[B16] Heinzen C. C. (2014). *Brain Plasticity in Speech Training in Native English Speakers Learning Mandarin Tones.* Master’s thesis, University of Minnesota Minnesota.

[B17] Johnson K. Mullennix J. W. (eds) (1997). *Talker Variability in Speech Processing.* San Diego, CA: Academic Press.

[B18] Linguistic Society of Hong Kong [LSHK] (2002). *Hong Kong JyutPing Character Table* 2nd Edn. Hong Kong: Linguistic Society of Hong Kong.

[B19] Lively S. E. Logan J. S. Pisoni D. B. (1993). Training Japanese listeners to identify English /r/ and /l/. II: the role of phonetic environment and talker variability in learning new perceptual categories. *J. Acoust. Soc. Am.* 94 1242–1255. 10.1121/1.408177 8408964 PMC3509365

[B20] Marslen-Wilson W. D. (1985). Speech shadowing and speech comprehension. *Speech Commun.* 4 55–51. 10.1016/0167-6393(85)90036-6

[B21] Nusbaum H. C. Morin T. M. (1992). “Paying attention to differences among talkers,” in *Speech Perception, Speech Production, and Linguistic Structure* eds Tohkura Y. Vatikiotis-Bateson E. Sagisaka Y. (Amsterdam: IOS Press) 113–134.

[B22] Peng G. (2006). Temporal and tonal aspects of Chinese syllables: a corpus-based comparative study of mandarin and cantonese. *J. Chin. Linguist.* 34 134–154.

[B23] Peng G. Wang W. S. Y. (2005). Tone recognition of continuous cantonese speech based on support vector machines. *Speech Commun.* 45 49–62. 10.1016/j.specom.2004.09.004

[B24] Peng G. Zhang C. C. Zheng H. Y. Minett J. W. Wang W. S. Y. (2012). The effect of intertalker variations on acoustic-perceptual mapping in cantonese and mandarin tone systems. *J. Speech Lang. Hear. Res.* 55 579–595. 10.1044/1092-4388(2011/11-0025) 22207701

[B25] Perrachione T. K. Lee J. Ha L. Y. Y. Wong P. C. M. (2011). Learning a novel phonological contrast depends on interactions between individual differences and training paradigm design. *J. Acoust. Soc. Am.* 130 461–472. 10.1121/1.3593366 21786912 PMC3155595

[B26] Sadakata M. McQueen J. M. (2013). High stimulus variability in nonnative speech learning supports formation of abstract categories: evidence from Japanese geminates. *J. Acoust. Soc. Am.* 134 1324–1335. 10.1121/1.4812767 23927129

[B27] Sadakata M. McQueen J. (2014). Individual aptitude in mandarin lexical tone perception predicts effectiveness of high-variability training. *Front. Psychol.* 5:1318. 10.3389/fpsyg.2014.01318 25505434 PMC4243698

[B28] Shinohara Y. Iverson P. (2018). High variability identification and discrimination training for Japanese speakers learning English/r/–/l. *J. Phonetics* 66 242–251. 10.1016/j.wocn.2017.11.002 23927129

[B29] So C. K. Best C. T. (2010). Cross-language perception of non-native tonal contrasts: effects of native phonological and phonetic influences. *Lang. Speech* 53 273–293. 10.1177/0023830909357156 20583732 PMC2897724

[B30] So C. K. Best C. T. (2014). Phonetic influences on English and French listeners’ assimilation of mandarin tones to native prosodic categories. *Stud. Second Lang. Acquis.* 36 195–221. 10.1017/S0272263114000047

[B31] Torkildsen J. K. Dailey N. S. Aguilar J. M. Gómez R. Plante E. (2013). Exemplar variability facilitates rapid learning of an otherwise unlearnable grammar by individuals with language-based learning disability. *J. Speech Lang. Hear. Res.* 56 618–629. 10.1044/1092-4388(2012/11-0125) 22988285 PMC3973537

[B32] Wang Y. Spence M. M. Jongman A. Sereno J. A. (1999). Training American listeners to perceive mandarin tones. *J. Acoust. Soc. Am.* 106 3649–3658. 10.1121/1.428217 10615703

[B33] Wang W. S. Y. (1972). “The many uses of F0,” in *Papers In Linguistics And Phonetics To The Memory Of Pierre Delattre* ed. Valdman A. (The Hague: Mouton) 487–503.

[B34] Wong P. C. M. Diehl R. L. (2003). Perceptual normalization for inter- and intra-talker variation in cantonese level tones. *J. Speech Lang. Hear. Res.* 46 413–421. 10.1044/1092-4388(2003/034) 14700382

[B35] Yip M. (2002). *Tone.* Cambridge: Cambridge University Press 17–208. 10.1017/CBO9781139164559.007

[B36] Ylinen S. Uther M. Latvala A. Vepsäläinen S. Iverson P. Akahane-Yamada R. (2010). Training the brain to weight speech cues differently: a study of finnish second-language users of English. *J. Cogn. Neurosci.* 22 1319–1332. 10.1162/jocn.2009.21272 19445609

[B37] Zhang C. C. Peng G. Wang W. S. Y. (2012). Unequal effects of speech and nonspeech contexts on the perceptual normalization of cantonese level tones. *J. Acoust. Soc. Am.* 132 1088–1099. 10.1121/1.4731470 22894228

[B38] Zhang K. Peng G. (2017). The relationship between the perception and production of non-native tones. *Proc. Interspeech* 1799–1803. 10.21437/Interspeech.2017-714 12597196

